# The Regulatory Roles of MicroRNAs in Bone Remodeling and Perspectives as Biomarkers in Osteoporosis

**DOI:** 10.1155/2016/1652417

**Published:** 2016-03-17

**Authors:** Mengge Sun, Xiaoya Zhou, Lili Chen, Shishu Huang, Victor Leung, Nan Wu, Haobo Pan, Wanxin Zhen, William Lu, Songlin Peng

**Affiliations:** ^1^Department of Spine Surgery, Shenzhen People's Hospital, Jinan University School of Medicine, Shenzhen 518020, China; ^2^Department of Orthopaedics and Traumatology, The University of Hong Kong, 21 Sassoon Road, Pokfulam, Hong Kong; ^3^Center for Human Tissues and Organs Degeneration, Shenzhen Institute of Advanced Technology, Chinese Academy of Sciences, Shenzhen 518055, China; ^4^Department of Orthopaedic Surgery, West China Hospital, Sichuan University, Chengdu 610041, China; ^5^Department of Orthopedic Surgery, Peking Union Medical College Hospital, Beijing 100730, China

## Abstract

MicroRNAs are involved in many cellular and molecular activities and played important roles in many biological and pathological processes, such as tissue formation, cancer development, diabetes, neurodegenerative diseases, and cardiovascular diseases. Recently, it has been reported that microRNAs can modulate the differentiation and activities of osteoblasts and osteoclasts, the key cells that are involved in bone remodeling process. Meanwhile, the results from our and other research groups showed that the expression profiles of microRNAs in the serum and bone tissues are significantly different in postmenopausal women with or without fractures compared to the control. Therefore, it can be postulated that microRNAs might play important roles in bone remodeling and that they are very likely to be involved in the pathological process of postmenopausal osteoporosis. In this review, we will present the updated research on the regulatory roles of microRNAs in osteoblasts and osteoclasts and the expression profiles of microRNAs in osteoporosis and osteoporotic fracture patients. The perspective of serum microRNAs as novel biomarkers in bone loss disorders such as osteoporosis has also been discussed.

## 1. Introduction

MicroRNA (miRNA) is a small noncoding RNA molecule (containing about 22 nucleotides) found in plants, animals, and some viruses, which also has functions in RNA silencing and posttranscriptional regulation of gene expression [1]. miRNAs interact with targets that have similar sequences, which inhibits translation of different genes, although miRNAs are not completely complementary to the mRNA sequence. miRNAs have been reported to impact on the expression of 1%~4% human genes by regulating about 30% human mRNAs [2]. Therefore, miRNAs play an important role in many biological processes, such as tissue formation [3–7], cancer development [8], diabetes [9], neurodegenerative diseases [10], systemic autoimmunity diseases [11], and cardiovascular diseases [12]. Interestingly, miRNAs have also been identified to be involved in the process of bone remodeling and many bone metabolic diseases, such as osteoporosis [13, 14].

The maintenance of bone mass mainly depends on the balance between osteoblast-mediated bone formation and osteoclast-mediated bone resorption [15]. Several miRNAs have been reported to regulate bone metabolism by modulating the differentiation and activity of osteoblasts and osteoclasts, such as miR-223 [16] and miR-103a [17]. Therefore, miRNAs are regarded as one of the important modulators in the bone remodeling. In this review, we will discuss the mechanisms of microRNAs involved in the bone formation and resorption, in particular the relationship between miRNAs and osteoblasts/osteoclasts. Furthermore, we present the recent reports on the expression profiles of miRNAs in osteoporosis and osteoporotic fracture patients. We hope this review will shed new light on the understanding of the characteristics of miRNA in bone remodeling and bone loss disorders.

## 2. Key Signal Pathways Involved in Bone Remodeling

**Figure 1:**
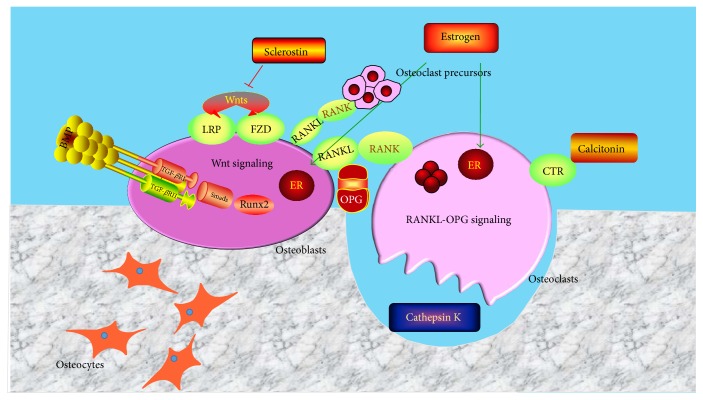
Bone cell interactions and key signaling pathways during bone remodeling. Osteoblasts induce RANKL and regulate the differentiation of osteoclast precursors. Meanwhile, the binding of RANKL and RANK stimulates the activity of osteoclasts. OPG produced by osteoblasts competitively binds to RANKL, resulting in the inhibition of osteoclasts differentiation. FZD and LRP form a coreceptor for Wnt ligand. The combination between Wnt ligand and its receptor results in the enhancement of osteoblast differentiation. Heterodimers of Ser/The form the receptor of BMPs. BMPs are regulated by Smads and Runx2.

Osteoblasts are differentiated from mesenchymal stromal cells (MSCs) through several key signaling pathways such as Wnt and BMP. Osteoclasts are originated from mononuclear precursors through RANKL-OPG signaling pathway. Although various cell types are involved in the process of bone remodeling, RANKL-OPG, Wnt, and BMP pathways have been identified as the classic pathways in the process of bone remodeling [18] (Figure 1).

### 2.1. RANKL-OPG Pathways

Osteoblasts and their progenitors are one of the key cell types in bone formation. Osteoblasts secrete numerous factors such as macrophage colony stimulating factor (M-CSF) and receptor activator of NF-*κ*B ligand (RANKL), which regulate the differentiation of osteoclast progenitors. Osteoclast progenitors finally generate mature osteoclast with markers, such as tartrate resistant acid phosphatase (TRAP) [19], calcitonin receptors [20], cathepsin K [21], and matrix metalloproteinase 9 (MMP9) [22], and alpha v beta 3 integrin chains (*α*_v_ *β*_3_) [23]. RANK is an important regulator of osteoclast precursors, along with CD14, CD11b, and cFms [24]. The binding of RANKL and RANK would affect many signaling pathways in the regulation of osteoclastogenesis, such as NF-*κ*B [25]. Osteoprotegerin (OPG) is a decoy protein secreted by osteoblasts and it could bind to RANKL, leading to the blocking of binding of RANKL and RANK and the inhibition of osteoclastogenesis. The RANKL-OPG pathway has been identified as a key regulator in the cross-talk between osteoblast-mediated bone formation and osteoclast-mediated bone resorption.

### 2.2. Wnt Pathways

The Wnt pathways are involved in bone homeostasis and bone remodeling process [26]. Wnts are regarded as anabolic factors that enhance the proliferation and differentiation of osteoblast progenitors [27–29]. Specific Frizzled (FZD) proteins and the low-density lipoprotein receptor related protein 5/6 (LRP-5/6) formed a receptor complex for Wnt ligand. With the binding of Wnt ligand, LRP-5, or LRP-6, *β*-catenin is released and become stabilized. Subsequently, *β*-catenin is translocated to the nucleus and regulates its target genes including Runx2 with the help of transcription factor 4 (TCF-4) or lymphoid enhancer-binding factor 1 (LEF-1) [30]. Additionally, Wnt signaling enhances osteoblast differentiation of bone marrow mesenchymal stem cells (MSCs) by increasing the level of Runx2 and osterix. On the other hand, Wnt signaling could suppress the differentiation of adipose-derived stem cells to adipocytes by inhibiting the adipogenic factors CCAAT/enhancer-binding protein *α* (C/EBP *α*) and peroxisome proliferator-activated receptor *γ* (PPAR-*γ*). Furthermore, Wnt signaling has impact on the suppression of osteoclast activity by secreted Frizzled-related protein 1 (Sfrp1), a binding site of Frizzled proteins. Sfrp1 competitively binds to RANKL, which reduces bone resorption [31, 32]. *β*-Catenin inhibits the level of OPG. Therefore, lower level of *β*-catenin in osteoblast progenitors resulted in bone loss [33].

There are also other regulators involved in the regulation of Wnt signaling pathways. For example, insulin-like growth factor 1 (IGF-1) interrupts the proteolytic degrading activity of *β*-catenin by affecting its stability and transcriptional activity [34]. Notch has been identified in the association to Wnt and acts by the degrading process of *β*-catenin [35]. Sclerostin, an antagonist of Wnt signaling, is found to be highly expressed in osteocytes by binding to LRP5/6. This process competitively affects the binding of Wnt and LRP5/6 [36]. Therefore, Wnt/*β*-catenin is a key signaling pathway for osteoblasts differentiation and activities.

### 2.3. BMP Pathways

Bone morphogenetic proteins (BMPs) are cytokines and belong to the members of transforming growth factor-*β* (TGF-*β*) superfamily. Heterodimers of Ser/The form the receptor of BMPs. BMPs are regulated by R-Smads (Samd1, Smad5, and Smad8) and Co-Smad (Smad4). The interaction between BMPs and their receptors stimulates the phosphorylation of Smads. The phosphorylated R-Smads form a complex with their common partner Smad4 and enter the nucleus to modulate gene expression and enhance osteogenesis [37].

Runt-related transcription factor 2 (Runx2), belonging to the Runx transcriptional factors family, connects many signal pathways in bone remodeling process by regulating osteoblast differentiation [38]. Runx2 regulates the gene related to bone formation such as osteopontin (OPN), bone sialoprotein (BSP) [39], and osteocalcin (OCN) [40]. It also enhances the level of PI3K/Akt [41]. Osterix, the downstream mediator for Runx2 actions during osteoblast differentiation, regulates the differentiation and function of osteoblasts and osteocytes, which is crucial for bone formation and bone resorption [42]. Runx2 is crucial in the activation of Smads by BMPs as well. Runx2 knockout mice exhibit defect in osteogenic activity [43]. Histone deacetylases (HDACs) are conserved enzymes that remove acetyl groups from lysine side chains in histones and other proteins and modulate gene expression in transcriptional corepressor complexes [44]. Among all HDAC family members, HDAC1 and HDAC3 are the most abundant in bone tissue [45]. HDAC5 bound to Smad3 in response to TGF-*β* and inhibited Runx2 activity [46]. HDAC9 was found to decrease in the process of osteoclastogenesis by inhibiting peroxisome proliferator-activated receptor gamma (PPAR*γ*) and receptor activator of nuclear factor kappa-B ligand (RANKL). In HDAC9 knockout mouse, the level of bone resorption was upregulated [47]. The fibroblast growth factors (FGF) pathway initiates the mesenchyme and proliferation of progenitor cells via transducing their signal through the FGF receptors (FGFRs). FGFRs contribute to normal skeletal development. The activation of FGFR2 signaling especially triggers increased Runx2 expression and enhanced osteoblast differentiation [48]. HOXA10 contributes to osteogenic lineage determination through activation of Runx2 and directly regulates osteoblastic phenotypic genes. In response to BMP2, HOXA10 is rapidly induced and functions to activate the Runx2, the transcription factor essential for bone formation. HOXA10 also activates other osteogenic genes, such as the alkaline phosphatase and osteocalcin [49]. FAK is not a necessary modulator for osteoblasts differentiation. However, once osteoblasts secrete their collagen type I, FAK-dependent integrin signaling appears to be critical for the structural protein to form an intact osteoid matrix [50].

## 3. Regulatory Roles of MicroRNAs in Bone Remodeling

miRNAs are endogenous noncoding RNA molecules and silence the transcription of mRNA by specifically binding to its target sequence. miRNAs play important roles during tissue development. In 2004, Chen et al. reported that miR-181 was strongly expressed in the B-lymphoid cells of mouse bone marrow [51]. Two years later, miR-140 was reported to be specifically expressed in cartilage tissue [52]. Cartilage is the major component of the skeletal system in early embryo stage. These studies indicate that miRNAs could be involved in the regulation of skeletal system. In particular, miRNAs have been reported to be involved in the modulation of osteoblasts and osteoclasts, two key regulators in the bone remodeling process.

### 3.1. Regulatory Roles of MicroRNAs in Osteoblasts

As a dynamic tissue, the maintenance of bone mainly depends on the balance between osteoblast-mediated bone formation and osteoclast-mediated bone resorption [15]. Osteoblasts are generated by bone marrow mesenchymal stem cells (BMSCs), synthesizing thickly, cross-linked collagen to constitute the major cellular ingredient of bone. From the morphological point of view, osteoblasts are cubical shaped cells with plenty of plasma. The cytoplasm of osteoblasts is in a strong basophilia state. Osteoblasts are found positive for periodic acid-Schiff staining and intensely positive for the alkaline phosphatase staining. Osteoblasts secrete osteocalcin and osteopontin, together with mineral to form bone matrix [53]. Osteoblasts continuously refill the bone cavity created by osteoclasts. The balance between osteoblast-mediated bone formation and osteoclast-mediated bone resorption is crucial to the bone remodeling and the homeostasis of bone metabolism.

Several specific miRNAs have been reported to be involved in the process of BMSCs differentiation into osteoblasts. For instance, miR-20a is expressed in many cell types and plays important roles in tumor proliferation and metastasis [54–57]. MiR-20a has also been identified to promote differentiation of BMSCs into osteoblasts. It directly binds to the 3′ untranslated region (3′ UTR) of BMP-2 [58] and activates BMP/Runx2 signaling through silencing peroxisome proliferator-activated receptors *γ* (PPAR*γ*), Bambi and Crim1 [59]. MiR-20a is also found to be overexpressed in osteoblasts derived from dexamethasone induction by downregulating RANKL expression, subsequently suppressing osteoclastogenesis and bone resorption [60].

MiR-29a is involved in regulating many cellular processes, such as the enhancement of T-cells stimulation [61, 62] and human immunodeficiency virus (HIV) replication [63, 64]. In skeleton system, the delivery of miR-29a inhibits the process of bone resorption in rats with glucocorticoid-induced bone loss [65]. Further* in vitro* study indicates that miR-29a mitigates bone loss by suppressing histone deacetylase 4 (HDAC4). This increased level of *β*-catenin acetylation and osteogenesis [66]. Additionally, the inhibition of HDAC4 expression could enhance Runx2 acetylation, which induce many growth factors and increase osteogenesis [67]. Furthermore, histomorphometric analyses indicate that miR-29a ameliorate the deterioration of trabecular bone volume caused by glucocorticoid in mice model. The bone mass of miR-29a transgenic mice is higher compared to that of wild-type mice [68].

MiR-15b regulates cell cycle progression and has been found to have close relationship with many kinds of cancer [69–71]. In bone remodeling process, miR-15b has been reported to play a positive role in the regulation of osteogenesis. In human BMSCs, miR-15b promotes the osteoblasts differentiation and shows a high amount of ALP and type I collagen. This process targets Smurf1 which protects Runx2 from degradation [72]. The expression level of miR-15b is also regarded as one of the criteria for the osteogenic evaluation [73].

MiR-210 is an important regulator in the setting of hypoxia [74–76]. It upregulates the expression level of vascular endothelial growth factor (VEGF) and enhances the differentiation rate of BMSCs into osteoblasts* in vitro* [77]. Another work completed by Mizuno et al. shows that miR-210 enhances the activity of activin-like kinase and the expression level of osteocalcin in ST2 cells by targeting activin A receptor type 1B (AcvR1b) gene [78].

MiR-216a shows a remarkable inhibitory effect on pancreatic tumor [79–81]. In human adipose-derived MSCs, miR-216a could significantly enhance their differentiation to osteoblasts by targeting c-Cbl. This induces phosphatidylinositol 3 kinase (PI3K)/AKT pathway in bone metabolism [82].

MiR-2861 has been reported to regulate the osteogenic differentiation of BMSCs. In mice, miR-2861 enhances osteoblasts differentiation by targeting histone deacetylase 5 (HDAC5) [83]. As HDAC5 deacetylates Runx2, the suppression of HDAC5 could elevate osteogenesis. MiR-3960 and miR-2861 are gathered at the same genetic locus. MiR-3960 regulates osteoblast differentiation through a modulation feedback loop with miR-2861 in primary mouse osteoblasts [84]. The target of miR-3960 is Homeobox A2 (Hoxa2) that is an inhibitor of Runx2. Additionally, the Runx2/miR-3960/miR-2861 regulation loop enhances osteogenic differentiation of vascular smooth muscle cells of mice with increased level of alkaline phosphatase (ALP) and osteocalcin (OC) [85].

It should be noted that miRNAs not only play a positive regulatory role in osteoblasts differentiation and activities, but also play a negative regulatory role. For instance, miR-204 inhibits osteoblast-related gene expression such as Runx2, osteopontin, and osteocalcin and decreased ALP activity of osteoblasts and bone matrix formation [86, 87]. Liao et al. demonstrated that miR-705 and miR-3077-5p suppressed osteoblast differentiation* in vitro* by inhibiting Homeobox A10 (HOXA10) and Runx2 mRNA, respectively [88]. Liu et al. demonstrated that miR-338-3P suppressed the expression level of osterix, a transcription factor for osteoblast differentiation in BMSCs by targeting Runx2 and Fgfr2 [89]. Moreover, resveratrol could prevent the bone loss in ovariectomized rats by inhibiting miR-388-3P [90]. MiR-125b shows a negative effect on osteogenesis by targeting osterix. The proliferation rate of hBMSCs is significantly decreased when transfected with miR-125b by MTT analysis. Meanwhile, qPCR results show that the levels of ALP, collagen *α*1, and osteocalcin were reduced as well, indicating that osteogenic differentiation was suppressed [91]. Interestingly, miR-637 shows an inhibitory effect on osteoblasts by targeting osterix as well [92]. MiR-188 has been reported to modulate the osteogenesis process by targeting histone deacetylase 9 (HDAC9) and RPTOR-independent companion of MTOR complex 2 (RICTOR). HDAC9 is a key factor that suppresses adipogenesis of BMSCs through PPAR*γ*. RICTOR, as a crucial member of mTORC2, also inhibits PPAR*γ* activity. The level of miR-188 in older mice is significantly higher than that in young mice. MiR-188 knockout mice exhibit reduced cortical bone loss [93]. MiR-141-3p suppresses osteoblast differentiation by targeting cell division cycle 25A in hBMSCs with decreased ALP and collagen [94]. MiR-138 has been reported to inhibit osteoblast differentiation by targeting the focal adhesion kinase (FAK). FAK activated ERK1/2 by the interaction with ECM proteins; the activated ERK1/2 further induces the phosphorylation of Runx2/Cbfa-1, affecting osteogenic gene expression [95]. Our group also found that miR-138-5p impaired skeletal cell proliferation and cell differentiation. We also found that miR-675-5p impaired skeletal cell proliferation, cell differentiation, and cell adhesion. MiR-675-5p induced MC3T3-E1 cell apoptosis more specifically than miR-138-5p [96].

### 3.2. Regulatory Roles of MiRNAs in Osteoclasts

MiR-503 has been reported to suppress osteoclastogenesis in CD14+ PBMCs by downregulating the expression of receptor activator of nuclear factor-*κ*B (RANK). The level of miR-503 is significantly lower in the serum of patients with postmenopausal osteoporosis than that of healthy control group. Additionally, micro-CT results of OVX mice femoral diaphysis show that the trabecular number is decreased with the addition of antago-miR-503 [97]. On the other hand, the positive effect of microRNAs on osteoclastogenesis has also been reported. For instance, miR-148a is upregulated during the process of osteoclasts differentiation. Microarray analysis showed a lower BV/TV of the trabecular bone measured in agomiR-148a-treated mice by *µ*CT indicating the positive effect of miR-148 on bone resorption* in vivo* [98]. Moreover, TRAP staining also reveals that miR-9 and miR-181a enhance osteoclasts survival rate [99]. Further study showed that the inhibition of miR-181a by estrogen could accelerate BMSCs-induced osteoclasts apoptosis [100]. The reported miRNAs involved in bone remodeling and their target genes are summarized in Table 1.

### 3.3. Dual Role of MicroRNAs in Bone Remodeling Process

MiR-34 family has been reported as cancer inhibitors by reflecting p53 pathway [108]. Interestingly, study also shows a higher level of osteogenic differentiation in mouse BMSCs with p53 deficiency [109], indicating the involvement of miR-34 family in bone remodeling process. MiR-34a inhibits the differentiation of osteoclasts by targeting transforming growth factor-*β*-induced factor 2 (TGF*β*-2). The miR-34a transgenic mice exhibit a higher bone mass [105]. On the other hand, miR-34c has been reported to inhibit the activity and differentiation of osteoblasts by targeting Notch1. In miR-34c transgenic mice, the value of bone volume/total volume, trabecular thickness, and trabecular number are significantly lower than that in wild-type mice, indicating that miR-34c suppressed osteoblasts differentiation [101].

MiR-26a has been demonstrated to positively regulate the differentiation of adipose tissue-derived stem cells (ADSCs) into osteoblasts by targeting SMAD1 transcription factor. SMAD1 interacts with the Homeobox C8 (Hoxc8) and leads to the activation of osteopontin gene transcription [104]. It is also confirmed by Wang et al's work that the scaffold containing miR-26a-modified ADSCs could enhance the potential of bone regeneration [110]. Interestingly, the modulation of miR-26a in BMSCs is different from that in ADSCs. MiR-26a in BMSCs targets GSK3*β* and Smad1 and regulates Wnt and BMP signaling pathway. The increased Wnt3a and BMP-2 improve mineralized nodule formation in BMSCs [111]. On the other hand, miR-26a has been reported to inhibit the osteoclastogenesis by targeting connective tissue growth factor (CTGF). TRAP staining results show that osteoclast formation was significantly suppressed by miR-26a [107].

MiR-214 inhibits bone formation by silencing activating transcription factor 4 (ATF4). In mouse preosteoblast MC3T3-E1 cells, with the treatment of miR-214 agonist, the levels of collagen I and ALP are significantly increased, showing the negative role of miR-214 in osteoblasts differentiation [112]. Meanwhile, miR-214 has been reported to enhance osteoclasts genesis from bone marrow monocytes (BMMs) via acting on PI3K/Akt pathway [106].

## 4. Current Biomarkers for Osteoporosis Treatment

In clinical setting, the established tools for evaluation of osteoporosis and bone fragility are bone mineral density assessment by Dual-X ray absorptiometry (DXA) and Fracture Risk Assessment Tool (FRAX), respectively [113]. However, DXA has several limitations. One limitation is that the assessment of BMD by DXA is based on the 2D, instead of 3D, images. The bone tissue properties, such as bone microstructure, which is essential for bone strength, cannot be measured by DXA. DXA also has quality control issues concerning errors from axial asymmetry of cross sections, assumptions used in calculating buckling ratios, and tissue mineralization assumption [114]. Meanwhile, since FRAX values the fracture risk with BMD criteria, it is not suitable for patients at high risk but without an osteoporotic BMD [115]. The risk of fracture is also influenced by other factors such as muscle function and the probability to fall. The parameters for evaluation of bone metabolism, such as beta C-terminal telopeptide (beta-CTX) and procollagen type I N-propeptide (P1NP), are important to assess the bone turnover and the response of the patients by the treatment. However, those parameters are not specific and not quite stable in the serum [116]. A single measurement of bone turnover could not estimate an absolute rate of bone loss. Moreover, they are not sensitive markers to reflect the bone fragility for those postmenopausal osteoporosis patients. It is, therefore, important to explore those stable biomarkers in the serum, which may be used for the detection of high risk of fracture in those postmenopausal osteoporosis patients. miRNAs are validated to be stable under harsh conditions including boiling, acid/alkaline circumstance, and freeze-thaw cycles with the help of high-density lipoproteins, which protectively bound to miRNAs from external RNases digestion [117]. Lipo-bound miRNAs are transported from the donor cells to the recipient cells by exosomes [118]. Meanwhile, the expression levels of serum miRNAs are reproducible and consistent among individuals [119], indicating that serum miRNAs could be suitable candidates for biomarkers in patients with osteoporosis.

## 5. The Potential Role of miRNAs as Therapeutic Targets

miRNAs have become an important therapeutic target for new drug research and development including analogs of miRNAs to enhance effectiveness and antagonist of miRNAs to interfere regulation, with advantages such as overcoming chemotherapy resistance, high efficiency, and low toxicity [120]. Clinical trial has been established in stage III colon cancer patients to study the anticancer effect by altering the binding capability of miRNA lethal-7 to Kirsten rat sarcoma viral oncogene homolog (KRAS) gene [121]. MiR-122 is an important target for the clinical study in hepatitis C patients. Long-term safety and efficacy study confirmed the effectiveness of targeting miR-122 therapy [122, 123]. Currently, clinical research on miRNAs involved in bone metabolism is still at the inception phase.* In vitro* and* in vivo* studies have shown a prospect on the clinical application of miRNAs for the treatment of osteoporosis. For example, miR-21 functionalized Ti implant could promote osteogenesis of BMSCs, which may provide a potential design for clinical use [124]. MiR-223 is reported to be an effective therapeutic target to relieve advanced glycation end products-induced damage to osteoblasts in diabetes mellitus [125]. MiR-150 could promote bone formation; the agomir of miR-150 may be considered as a novel therapy for osteoporosis [126]. MiR-214 is a key regulator of osteoclast differentiation, which may be considered as a promising therapeutic target for osteoporosis [106]. However, the major concern for the application of miRNAs in osteoporosis treatment is the difficulty in delivering miRNA into the cells, which has a great impact on the efficacy of miRNAs.

## 6. MicroRNAs Expression Profiles and Perspectives as Biomarkers in Osteoporosis

As the evidence for the important role of miRNAs in the regulatory of bone remodeling is increasing, the association between miRNAs and bone loss disorders such as OP has been investigated by several groups. On one hand, the expression profile of serum miRNAs in the postmenopausal women has been studied. For instance, the expression of miR-422a and miR-133a in the serum is significantly different between postmenopausal women with low bone mineral density (BMD) and those with high BMD [127, 128]. The expression of miR-21 is significantly higher in the serum in OP patients compared to the control [129]. On the other hand, the expression profile of miRNAs in the patients with osteoporotic fractures has been investigated. For example, Panach et al. examined the plasma microRNAs from postmenopausal women with or without osteoporotic hip fractures and found that miR-122-5p, miR-125b-5p, and miR-21-5p were significantly upregulated in the patients with osteoporotic hip fractures compared to those without osteoporotic hip fractures [130]. This work is consistent with the previous work published by Seeliger et al., who demonstrated the significantly different expression level of miR-21, miR-23a, miR-24, miR-25, miR-100, and miR-125b in patients with hip fracture [131].

**Figure 2:**
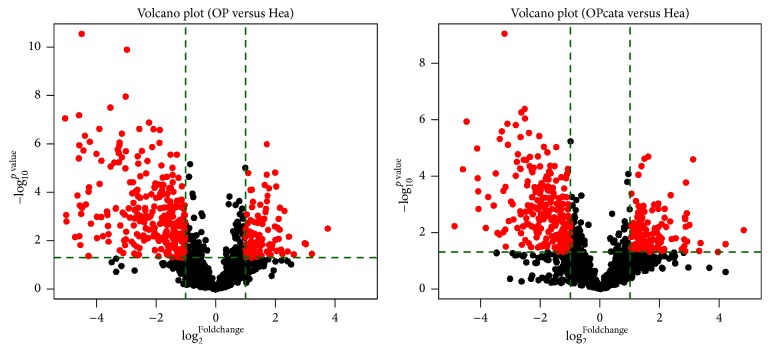
The volcano plot of miRNAs within each group. The red dots represent the miRNAs which were significantly differently expressed with *p* values < 0.05 and foldchange values ≥ 2 or ≤ 0.5.

Our research group has also explored the expression profile of serum miRNAs in the postmenopausal osteoporotic women with or without vertebral fractures and the control. We have found that several miRNAs such as miR-106b and miR-19b were significantly different either between OP group and control or between OP with vertebral fracture group and the control group (Figure 2).

## 7. Summary and Future Directions

miRNAs are endogenous molecules in our body and possess specific properties including easy access, high stability, tissue specificity, and significant sensitivity. They target specific genes and emerge in bone resorption and formation. This is also associated with many bone disorders. However, the mechanism and potential application still remain elusive. To elucidate this, there is at least three important points to be identified and clarified. Firstly, what are the specific miRNAs in bone remodeling? It is necessary to distinguish two groups of miRNAs involved in bone resorption and formation, respectively. This will pave important way for further study. Secondly, what is the significant expression level in the bone remodeling? miRNA is always minimally expressed and it has not been quantified to be in standard. Thirdly, how to detect the key miRNAs in bone tissue? Combined with biochemistry and special molecular technology, it is feasible to identify the expression profile of miRNA in peripheral circulation; however, further consideration is needed to implement the detection in bone tissue. Altogether, the miRNA will propose novel understanding for the bone biology and treatment.

## Figures and Tables

**Table 1:** The current research related to miRNAs involved in osteoblast and osteoclast differentiation.

MicroRNA	Effect	Target	Reference
MiRNAs related to osteoblasts differentiation			
MiR-210	Upregulate	VEGF	[77]
MiR-216a	Upregulate	Cb1-mediated PI3K/Akt	[82]
MiR-29a	Upregulate	Runx2	[68]
MiR-20a	Upregulate	BMP/Runx2	[59]
MiR-204	Downregulate	Runx2	[86]
MiR-705	Downregulate	HOXA10	[88]
MiR-3077-5p	Downregulate	Runx2	[88]
MiR-103a	Downregulate	Runx2	[17]
MiR-34c	Downregulate	BMP2	[101]
MiR-17-5p	Downregulate	BMP2	[102]
MiR-106a	Downregulate	BMP2	[102]
MiR-125b	Downregulate	Osterix	[91]
MiR-637	Downregulate	Osterix	[92]
MiR-188	Downregulate	Histone deacetylase 9 (HDAC9) and RPTOR-independent companion of MTOR complex 2 (RICTOR)	[93]
MiR-141-3p	Downregulate	Wnt signaling pathway	[94]
MiR-138	Downregulate	Focal adhesion kinase signaling pathway	[95]
MiR-338-3P	Downregulate	Runx2 and Fgfr2	[89, 90]
MiR-2861	Upregulate	HDAC5	[83]
MiR-378	Upregulate	Caspase-3	[103]
MiR-26a	Upregulate	SMAD1	[104]

MiRNAs related to osteoclasts differentiation			
MiR-503	Downregulate	RANKL	[97]
MiR-148a	Upregulate	RANKL	[98]
MiR-34a	Downregulate	TGF*β*-2	[105]
MiR-214	Upregulate	PI3K/Akt	[106]
MiR-17	Downregulate	RANKL	[60]
MiR-20a	Downregulate	RANKL	[60]
MiR-26a	Downregulate	CTGF	[107]
